# Ophthalmology

**Published:** 1913-09

**Authors:** Cyril H. Walker


### OPHTHALMOLOGY.

**Toxæmia (Alimentary and Oral) as a Cause of Eye Disease.**—Evidence is accumulating that almost all inflammatory diseases of the eyes may be due, directly or indirectly, to auto-intoxication from the mouth or intestines. There is a good epitome of recent literature of this subject in *The Ophthalmoscope* of July, 1913. Amongst the cases referred to we find acute retrobulbar neuritis affecting both eyes, unilateral irido-choroiditis leading to complete blindness in infants suffering from acute gastro-enteritis, and Risley, of Philadelphia, to this cause attributes many cases of phlyctenular ophthalmia. He also quotes cases of optic atrophy, neuro-retinitis with increased blood pressure, and choroido-retinitis with papillitis due to auto-intoxication. Lawford[3] and most other authorities look upon irido-cyclitis as being in many instances due to pyorrhœa alveolaris, and diseases of the corneo-sclera and uveal tract are looked upon as being specially likely to be the result of auto-intoxication. B. T. Lang[4] gives an analysis of 176 cases of inflammation of the eye, in 71 of which the trouble was referable to buccal sepsis. He points out that inflamed mucous areas, such as the gums, which are subject to frequent mechanical disturbance, are the most likely to give rise to eye troubles. It is often argued that pyorrhœa is excessively common, whilst irido-cyclitis and

[3] *Proc. Roy. Soc. Med.*, May, 1913 (*Suppl.*) p. 121.

[4] *Brit. M. J.*, 1913, i. 381.

similar diseases are comparatively rare. W. Lang[1] points out that this only implies that the protecting barrier raised by Nature is generally efficient. He rather questions the wisdom of the dental profession in crowning teeth and building bridges that cannot be kept clean. When Nature's protecting barrier has broken down, and the eyes are affected by a serious inflammation, it is then too late to do anything except to remove the teeth at once. He thinks that the possibility of a septic focus should be investigated, and if found, treated in all cases before an operation on the eyeball is performed. Central senile choroiditis is, in Lang's opinion, no more caused by old age than is the senile edentulous jaw ; " both are due to pyorrhœa, and if the mouth were looked after from youth onwards two of the attributes of old age —*sans teeth, sans eyes*—would not occur."

If alimentary toxæmia is at all a frequent cause of eye disease its early recognition is most important. Elschnig thinks that indicanuria in eye disease is evidence of gastro-intestinal auto-intoxication, and that the latter is a probable cause of the disease. Stuelp, of Mülheim, investigated 82 cases of irido-cyclitis without finding any evidence of indicanuria. Von Hippel, of Halle, in 416 cases found indicanuria in only 16. Colombo, of Parma, however, supports Elschnig's view, and emphasises the importance of testing the urine early before the institution of treatment. His cases show that the indican reaction may disappear with the improvement of the eye condition and return during relapses.

S. H. Browning[2] advocates the routine examination of the fæces and urine, at any rate in those cases in which the cause of the eye disease is not immediately obvious. The pyorrhœa alveolaris may have been cured, leaving a general infection of the alimentary tract as a result of many years of pus swallowing. Such patients with chronic irido-cyclitis may have clean mouths, but on examination of the fæces may show that they are suffering from bacterial infection of the bowel. He has found other patients with colitis, perhaps unsuspected, or only suffering from chronic constipation, who have been cured of the eye disease by vaccine and other treatment.

The proof that the eye condition is caused by the intestinal or urinary infection is the cure of the eye disease at the same time as the other general condition. Browning finds this can most readily be brought about by the use of an autogenous vaccine.

**The Causation of Myopia.**—Myopia is often regarded as an over-adaptation of the eye to the requirements of civilisation, but it must not be forgotton that hypermetropia, in England

[1] *Lancet*, 1913, i. 1368. [2] *Ophthal. Rev.*, April, 1913.

at any rate, is still about five times as common as myopia. Most are agreed that in a great many instances myopia is associated directly or indirectly with school-work, and great pains are being taken to prevent the occurrence of myopia in schools, and by means of special classes to mitigate its extent and retard its progress in those instances where it has already become manifest. The exact relationship between school-work and myopia is, however, still in dispute. Sattler in 1907 attributed myopia to the pressure of the recti muscles upon the eyeballs when the movements of the eyes necessitated in reading are produced. The consequent increase of intra-ocular tension, according to him, stretches the posterior segment of the globe. But there is no evidence that the intra-ocular tension is increased by the action of the recti ; secondly, where (as in buphthalmos) the intra-ccular tension is known to be increased, there is no yielding of the posterior segment ; thirdly, the changes in myopia are quite unlike those found in increased tension.

Wilson [1] reviews 500 cases of myopia which have come under his notice, 338 females and 162 males. Hereditary tendency to myopia was found in 54 per cent. Keratitis, or some similar inflammation of the anterior segment of the eye, appeared to be the next most important factor, since nearly 20 per cent. had some degree of corneal opacity. He points out that the near work of school years is contemporaneous with manifestations of heredity, and that it is possible that the respective responsibilities of each may be confused. He regards myopia as essentially an enfeeblement of the resisting structures of the posterior segment of the eye, and thinks that keratitis in some way—perhaps by inflammation spreading backwards—affects the resisting power of the sclera. In his view, it is not so much the school-work and conditions which should receive attention as the local health of the eye and the general vigour of the body which should be improved.

Harrison Butler, reviewing Levinsohn's book on myopia, [2] draws attention to facts which rather support this view. During the last fifty years the frequency and degree of myopia amongst medical students in Germany have not altered. For fifty years Germany has paid great attention to school hygiene, and yet the proportion of myopia is not reduced. It is quite likely that any cause which makes the body flabby will increase the undue distensibility of the sclera. In Sweden, where *games* in the open air have replaced drill, the percentage of myopia amongst students has fallen in a most astonishing manner. Levinsohn holds the view that in looking down, as for instance in reading, the eyes are to some extent suspended in the orbits by the optic nerves, and that the tension on the posterior segment of the eye causes the sclerotic to yield. This theory is open to many

[1] *Glasgow M. J.*, 1913, lxxix. 250. [2] *Ophthalmoscope*, 1913, xi. 54.

objections, amongst others that myopia, even in high degree, is found in children of three or four years of age, and that it is common to find one eye highly myopic whilst the other is emmetropic.

On the whole, for practical purposes, it seems best to adopt the view that myopia is due to enfeeblement of the posterior segment of the eye, and to aim at improving the physique of the patient, as well as the conditions of school work.

**Sympathetic Ophthalmitis.** — Franz Deutschmann, of Hamburg, claims to have produced sympathetic ophthalmitis in monkeys by introducing pieces of choroid from cases of the disease in man. He also claims to have found a Gram-positive diplococcus, which is itself capable of producing sympathetic ophthalmitis. His experiments go to prove that the track taken by the micro-organism from the exciting eye to the sympathising eye is from the choroid into the orbital lymphatics, and from them into the optic nerve-sheath, and so via the chiasma into the nerve-sheath of the other eye. From this it reaches the lymphatics surrounding the second eye, and eventually attacks the choroid. Deutschmann's results are, however, disputed. Some regard the experimental sympathetic ophthalmitis as being only a uveitis caused by the introduction of alien albuminous matter.

CYRIL H. WALKER.

